# Rhinovirus infection has allergen-specific tolerance-breaking effects on PBMCs of healthy individuals

**DOI:** 10.1186/1939-4551-8-S1-A257

**Published:** 2015-04-08

**Authors:** Umut Can Kucuksezer, Alar Aab, Beate Rueckert, Gunnur Deniz, Cezmi Akdis, Mübeccel Akdis

**Affiliations:** 1 Istanbul University, Institute of Experimental Medicine (DETAE), Turkey; 2 Swiss Institute of Allergy and Asthma Research (SIAF), Switzerland

## Background

Allergen-specific T cell tolerance is important in healthy immune responses to environmental allergens, mechanisms of loss of tolerance to allergens is still not completely understood. Studies investigating maintenance and breaking of peripheral T cell responsiveness clearly showed the contribution of inflammatory cytokines and triggering of TLR4 and TLR8 to loss of unresponsiveness to allergens. Human rhinoviruses are most common viral infective agents in humans and the predominant cause of cold. The primary route of entry is the upper respiratory tract. Upon infection, virus replicates, spreads and cause infected cells to release chemokines and cytokines. Rhinovirus infections are known to be associated with an increased risk of asthma development, and among children with prevalent asthma, 85% of asthma exacerbations are associated with viral infections. However, the exact nature of this relationship remains unclear.

## Methods

PBMCs of healthy individuals with known healthy responses to allergens were incubated with Rhinovirus 1B and 16 strains, with increasing doses with the absence or existence of allergens in cell-culture conditions. On day +5, cell proliferation was investigated with (3H)-thymidine incorporation or CFSE dilution methods. Rhinovirus strains were produced upon infection with HELA cells and culture supernatants containing alive rhinovirus was used in cultures. TCID50 of viral infection was evaluated and dilutions in PBMCs were made according to this value.

## Results

Our results demonstrate the allergen-specific T cell tolerance-breaking effects of both strains in different virus doses.

## Conclusion

More detailed studies are needed to clarify molecular mechanisms for breaking of allergen-specific unresponsiveness by rhinovirus infection.

